# On Sectional Medicine

**Published:** 1844-11-02

**Authors:** Henry W. Bullitt

**Affiliations:** Louisville, Kentucky


### THE MEDICAL EXAMINER,

### And Record of Medical Science.

Vol. VII.] PHILADELPHIA, SATURDAY, NOVEMBER 2, 1844. [No. 22.

### ON SECTIONAL MEDICINE.

By Henry W. Bullitt, M. D., of Louisville, Kentucky.

Several years since, the position was taken by a writer for a medical periodical, published at Louisville, Ky., that the diseases prevalent in the Valley of the Mississippi, were so entirely unlike those which present themselves in the North Eastern states of the Union, as to make it necessary for the physician to study his profession in whichsoever of these sections of country he designed to practise. It was contended, by this writer, that a physician educated at Boston, New York, Philadelphia, or Baltimore, would become imbued with pathological and therapeutic notions, which would be fatally pernicious, if practically applied, at any locality in the South Western States.

This position, although presenting itself then for the first time, so far as we know, on the pages of a scientific journal, was not new to us; for we had combated it, time and again, in private circles, and had heard it advanced and advocated in the halls of the Louisville Medical Institute. We were not, therefore, at all surprised when we discovered that it had found its way into the pages of a Journal, the recognized organ of the Institution, in the halls of which we had so often heard it orally promulgated.

The doctrine was so utterly at variance with what we considered the established principles of medical science, and the arguments advanced in support of it were so pointless and unsatisfactory to our mind, that we did not, at the time, deem it worthy the trouble of a formal refutation. We regarded it as a sort of "brutum fulmen," contrived and launched forth by the institution, from which it emanated, to deter the Medical Students of the Mississippi Valley from crossing the mountains to complete their professional education. And, although we did not approve of the device, we were yet willing that its authors should enjoy the little advantage likely to accrue from it to their young and flourishing institution; because we could not bring ourself to believe that any persistive effort would be made to give extended circulation to a heresy in science, so obviously untenable, and so pregnant with mischief. We hoped that those even, with whom the thing originated, had too high a regard for the sanctity of science, and for the honour of their calling, to continue long to nurture a sentiment so entirely irreconcilable with all pretention on the part of medicine to the character and dignity of a science. But in this expectation we have been disappointed. The heresy has not been abandoned. On the contrary, its authors have persisted in it, and in the true spirit of champions, strong in faith and determined to triumph, have they advocated it. Article has followed article, now in the medical Journal, and now in political newspapers, written all in the most approved style of the trained polemic. Dogmatism and intolerance, as well as sophistry, pervade them throughout. Nowhere do they present evidences of self-forgetfulness, the charity and the candor which are invariable characteristics of the writings of all those who seek truth for its *own sake*.

The following is an extract from one of these articles, and is a fair specimen of the style and spirit of the whole of them, as well as a full and frank expression of the nature and purport of the doctrine in question.

"The Professors of the Atlantic Schools are but very moderately qualified, or rather, not qualified at all, to communicate to their pupils such views of practical medicine as are suited to the diseases of the Mississippi Valley. How, indeed, can the case be otherwise? How is it possible for those professors, however learned and able they may be, to teach successful modes of treating diseases which they have never seen, and of whose ruling and peculiar features and characters they are necessarily uninform d. In fact, they neither do possess, nor can possess, such resources for teaching; nor can they ever attain them, unless they pass the mountains and spend years in the midst of us, observing, studying and treating our complaints. Let them thus act, and thus accomplish themselves, or else confine their instructions to the cure of maladies with which they have been conversant, and detail them only to medical pupils of their own region. For practical purposes, in the West, those instructions have neither fitness nor value." This extract developes the doctrine of the writer fully and fairly, and we have given it thus at length, that we may have no chance of misrepresenting it. We desire to treat it with all fairness and candor; for, if we know ourself, truth is our object, and this could not be gained by misrepresentation.

From the earliest period of our professional studies, although educated in the West, we were taught to regard medicine in the light of a science, founded, like all the "natural sciences," upon fixed and immutable principles. That it is not, at present, entirely certain in all of its ongoings is the fault, not of the science itself, but of the difficulties that beset its cultivation. The medical philosopher, in his investigations, has to deal with the most refined and intricate operations of nature: and, what adds immensely to the difficulties of his task, there is scarcely a link in the whole circle of natural existence which it is not his province to scrutinize. With the properties of matter, in whatever form it presents itself, he is required to be familiar. Every material entity, from the inorganic element, up to the noblest of organized beings, is an object of peculiar interest to him; not alone, on account of its physical qualities, but more especially in reference to its dynamic or vital properties.

With this vast and almost boundless field of inquiry to cultivate, it is only surprising that he has done so much towards establishing the claims of medicine to its place in the category of the natural sciences. In the department of natural philosophy, technically so called, and in that of Chemistry, a degree of certainty has been attained, which justly ranks them with the exact sciences. And the studies of Zoology and Botany have been so successfully prosecuted, as to give them indisputable claims to a like consideration. Nor can it be denied, that anthropography, or the science which treats of the anatomy and physiology of man, is sufficiently certain, in most of its details, to entitle it to a similar rank. It is true we as yet know but little of transcendental or molecular anatomy, and less perhaps of many of the more complete phenomena of life. But it is equally true, that wherever the observation of particular facts has been sufficiently accurate and extensive, we have had revealed to us laws as fixed and as unvarying in their operation, and as faithful in their results, as are those which give character to the most certain of the sciences; and what especially concerns us at the present time, it is not less true, that those laws are alike efficient and uniform in their operation throughout the world. No one who has any regard for the dictates of common sense, can contend, that Meckel's Manual of Anatomy or Müller's Physiology are less valuable as works of science in Philadelphia and Baltimore, than in the country of their origin; neither can it be successfully maintained, that the principles which they embody are less applicable in the Valley of the Mississippi, than along the shores of the Atlantic ocean. It is admitted, we believe, by all enlightened anatomists, that the principle of the "Unity of Organic Composition," as taught by St. Hilaire, is authorized and sustained by all that we know of the intimate structure of organized beings. Were it not, indeed, true that the organic elements, under whatever form they present themselves, are always the same, nothing of a philosophical, or truly scientific character could attach to Anatomy. Did we base its claims to the rank of a science upon the varying and fugitive forms of the organs, the exceptions would soon become too numerous for the rule. But when we extend our view beyond the mere conformation of the organs into their interior, and make the elements themselves the objects of investigation, we discover a simplicity of composition, and a uniformity of arrangement, of which the mere special anatomist can have no conception, and what is more pertinent to our present inquiry, we also discover, that these elements of composition are every where the same, and that they are uninfluenced in their formation and arrangement, by any of the varying conditions of climate, or of geological formation. Indeed, so universal and fixed is the belief amongst sound medical philosophers, that the healthy conditions of organic compositions and of organic action, are every where, and under all circumstances, identical, that it would be a work of supererogation to say more in support of this doctrine. Already has it been made the basis of the most philosophical explanation of the various congenital monstrosities that has ever been given. By means of it these singular phenomena, which, in former and darker days, were attributed to the agency of demons, or to the wrath of the Deity, are now classified and explained as satisfactorily as any other natural phenomena. And, certainly, no one will contend that the classification of "Monsters" by G. St. Hilaire, Serres and Meckel, and the theory by which their formation is accounted for, are less worthy of confidence here in the Valley of the Mississippi, than on the continent of Europe. They are, like the laws upon which they are based, fixed and immutable, and of universal applicability.

The discovery of the fact, that the intra-uterine deviations from the normal condition of the organs resulted from the operation of fixed and appreciable laws, led naturally enough to the inference, that similar deviations from the healthy state, which occur after birth, might also observe fixed rules in their developement. Accordingly, we find all the ablest pathologists of the present time advancing and maintaining successfully, as we think, the opinion that all the morbid actions of the organs progress in obedience to laws as uniform in their operation as are those which preside over their healthy actions. "That morbid, like healthy action, has its laws, and that disease, in many of its apparently fickle changes and protean forms, is but proceeding in obedience to certain rules, are positions established by innumerable examples, and by convincing proofs." Thus writes Colles, an able pathologist of Dublin; and were this not the case, what would the laborious researches of Andral, Cruveilhier, and Louis, of Hunter, Carswell and Hodgkin, of Stokes, Graves and Corrigan avail us in America? Would it not be absurd to fill our libraries with the books of these writers, were they replete with principles and doctrines totally inapplicable to our diseases? Would not experience, sad, fatal experience, long since have banished them, together with the works of all their predecessors from the library of every American physician? Would not our schools of medicine have ceased, long since, to use as their text books, the works of European authors? These questions cannot be negatively answered; and the fact, that they cannot, should be sufficient to satisfy every reasonable mind of the correctness of the declaration of Colles.

But it may be admitted that morbid action has its rules, and at the same time contended, that these rules are entirely different in different geographical positions. The fact just stated, that the opinions of European observers are regarded as high authority in America, would meet this difficulty also. But we shall not meet it thus. It is a question of *fact*, and should be treated as such.

It cannot be denied that all the epidemics which, at different periods, have spread themselves over the habitable globe, have advanced from place to place uninfluenced ostensibly by climate or geological formation. The influenza of 1792 spread itself from China, where it originated, over all the inhabited parts of Asia, Europe and America, and everywhere presented the same phenomona, and called for like modes of treatment. This was also the case with the influenza of 1837. Of this latter epidemic Dr. Graves says, that for several weeks before it made its appearance at Dublin, he was familiar with its symptoms and the best method of treating it. And he farther states, that he had acquired from eastern authors a thorough familiarity with the symptoms of Asiatic cholera, and was prepared to treat it several years before he witnessed a case of it.

But it is not alone in the case of wide spreading epidemics, that pathological and therapeutic laws are uninfluenced by known physical agents. It has never been doubted, for instance, that all the simple phlegmasiæ proceed in obedience to known and uniform laws. The knowledge of this fact is the basis of all surgical operations. Were it not known to the operator that inflammation always succeeds the use of the scalpel, and advances in accordance with established rules, no sane and conscientious man could ever think of undertaking any of the numerous operations, the successful conduct of which is daily reflecting so much glory upon the profession, and rescuing so many valuable lives from a premature end. Neither has it been suggested, as far as we know, by any one, that any of the diseases called specific, as variola, vaccinia, syphilis, &c., or of those known as malignant, such as scirrhus, and the various heterologous formations, are influenced materially in their essential features by geographical position. Indeed, we think we may safely narrow down this whole controversy to the class of diseases known by the general designation of fever; for even those who contend most strenuously, and in the most general terms, for the peculiarity of western and southern diseases, have never specified any other forms of diseases as presenting these peculiarities.

The varieties of fever met with in the United States may be classed under the heads of: 1st. Intermittent; 2d. Remittent; 3d. Continued. Of the Intermittent varieties, we have the simple and the complicated. Of the Remittent, we have numerous modifications, including, according to most authorities, the "Yellow Fever." Of the proper Continued fever we have only the Typhus and the Typhoid.

The typhus fever and the typhoid affection have been ascertained by adequate and satisfactory observation, to be the same in their essential characters, and to require like modes of treatment wherever they have occurred; and they have been observed in almost every part of the habitable globe. The typhoid affection, so elaborately described by Louis as the prevailing fever of Paris, has been observed in every latitude in the "United States," and the descriptions given of it by American physicians have enabled the Parisian professors to recognize it without difficulty as the true dothinenteritis of that metropolis. And from Dr. Gerhard's account of an epidemic of typhus fever, observed in Philadelphia during the winter of 1836–37, it will be found that this malady presented the same features in that city which have characterized it throughout Great Britain and on different parts of the continent of Europe. We may, therefore, for the present consider it as a settled proposition, that the petechial typhus fever and the typhoid affection are the only proper continued fevers, and that they observe, in their origin and progress, the same laws and present the same therapeutic indications wherever they have been witnessed.

In reference to intermittent fever we need not say much. It can scarcely be denied, that the uncomplicated varieties are the same in all latitudes. Certainly, in no part of the world, do enlightened physicians undertake to treat this disease without the aid of some of the preparations of bark; so that, let the theory be what it may, the treatment is the same every where. With regard to the complicated varieties, we will only remark, that the complications which alone constitute the differences between the milder and more grave forms of the disease, consist of modes of morbid action with which physicians in all parts of the world are familiar as distinct maladies. This being the case, there can be no good ground for the opinion, that the complicated intermittents of the southwestern states are so unlike the simpler forms of temperate latitudes, as to make special courses of instruction necessary to enable the physician to treat the one or the other.

We have thus narrowed down, by what we consider legitimate argument, the whole of this controversy to the simple question, whether the "*remittent fevers*" of the United States present the much talked of sectional peculiarities? Remittent fever prevails along the shores of the Northern lakes—it prevails, more or less, throughout the state of New-York; the cities of Boston, New-York, Philadelphia and Baltimore, are not exempt from it—it is encountered along the banks of the Mississippi and of all its tributaries. Now does it, in these different localities, present such marked peculiarities, as to make special courses of study necessary to enable physicians to practise in one or another of these various situations?

We have taken the trouble to examine the accounts, given by different writers, of the yellow fever, as it prevailed during the latter part of the last century and the beginning of the present, in Boston, New-York, Philadelphia and Baltimore; and we have compared these with the accounts given by southern writers, and especially with that given by Louis, of the epidemic observed by him at Gibraltar, and that given by Rufz of the disease as witnessed at Martinique, and we have not been able to discover any differences of importance in any of these descriptions, nor in the principles of treatment. Indeed we presume it will not be contended by any one, that the yellow fever of New-Orleans or Mobile is at all different from the yellow fever of the eastern cities as observed during the period above indicated. In reference to the other varieties of remittent fever, we might cite the essay of Dr. Stewardson of Philadelphia on the subject, and defy all those who contend for the peculiarity of southwestern fevers to produce anywhere a disquisition containing clearer or more pertinent views of the pathology of these fevers, or rules of treatment more in accordance with that adopted by the most judicious practitioners of the west. We do not regard this essay as a full and satisfactory account of our remittent fevers; but we consider it as good as any we have seen. These fevers have not as yet been thoroughly investigated, and until this is done, it cannot, of course, be successfully maintained, that they present decided pathognomonic differences in different sections of country. On the contrary, we confidently appeal to all that has been written in relation to them, to sustain us in the adverse opinion, that so far as they have been accurately observed, they have been found exhibiting remarkable similarity, if not absolute identity, in all of the various localities of the union. We should have preferred discussing this subject in its connexion with fevers, by making copious extracts from the various writings of western and southern practitioners, and comparing them with the corresponding productions of the eastern writers; but the limits prescribed for our remarks, will not permit us thus to discuss it at present. By this means, we think we should have been able to show, that even those who talk most loudly about *sectional peculiarities* have failed to indicate them in their accounts of the very diseases which they contend present them in the most striking manner. Indeed we might indulge to almost any extent in this sort of "argumentum ad hominem," but by it we should prove nothing, though we might confound our adversaries. We might, for instance, dwell upon the fact, that in the Louisville Medical Institute, the "Text Book" of Pathology is a work of western origin, and that its prominent feature is the long since exploded doctrine of Broussais, that all disease consists in inflammation,—a doctrine totally at variance with the one we have been combating; which would make new diseases for every degree of latitude. We might refer to the fact, that the text-book of theory and practice, in the same Institution, is a work of British paternity, and that not one of the text-books in any of the western schools, with the single exception just indicated, boasts a western origin. We might, also, inquire, how it is that the schools of Kentucky and Ohio are suitable places of instruction for those who design practising in Mississippi and Louisiana? since the difference of the climate between these latter states, on the one hand, and Kentucky and Ohio, on the other, as also the differences of geological formation, are fully as great, if not greater, than are those which exist between the climate and geology of Pennsylvania and Kentucky. But in this description of argument we forbear to indulge at present, satisfied if we have succeeded in presenting the legitimate arguments on the subject in such manner as to induce reflection.

In conclusion we would have ourself distinctly understood as placing too high an estimate on the teaching talent of the west to suppose that it needs the aid of any such flimsy pretence, as that of the peculiarity of western diseases, to secure it its full meed of enlightened public patronage. We have teachers in some of our schools of medicine, who will compare most advantageously with the ablest in the schools of the Atlantic cities. Let them, therefore, maintain an honorable competition with their eastern brethren. Let them take no advantage other than such as their talents and industry entitle them to; and let the public determine upon the comparative merits of eastern and western schools by the accomplishments and success of their respective alumni.

